# Upregulation of the receptor-interacting protein 3 expression and involvement in neural tissue damage after spinal cord injury in mice

**DOI:** 10.1186/s12868-015-0204-0

**Published:** 2015-10-08

**Authors:** Haruo Kanno, Hiroshi Ozawa, Satoshi Tateda, Kenichiro Yahata, Eiji Itoi

**Affiliations:** Department of Orthopaedic Surgery, Tohoku University School of Medicine, Seiryo-machi, Aoba-ku, Sendai, Miyagi 980-8574 Japan

**Keywords:** Receptor-interacting protein 3, Spinal cord injury, Necroptosis, Cell death, Necrosis, Apoptosis

## Abstract

**Background:**

Necroptosis is a newly identified type of programmed cell death that differs from apoptosis. Recent studies have demonstrated that necroptosis is involved in multiple pathologies of various human diseases. Receptor-interacting protein 3 (RIP3) is known to be a critical regulator of necroptosis. This study investigated alterations in the RIP3 expression and the involvement in neural tissue damage after spinal cord injury (SCI) in mice.

**Results:**

Immunohistochemical analysis demonstrated that the RIP3 expression was significantly increased in the lesion site after spinal cord hemisection. The increased expression of RIP3 started at 24 h, peaked at 3 days and lasted for at least 21 days after hemisection. The RIP3 expression was observed in neurons, astrocytes and oligodendrocytes. Western blot analysis also demonstrated the RIP3 protein expression significantly upregulated in the injured spinal cord. RIP3 staining using propidium iodide (PI)-labeled sections showed most of the PI-labeled cells were observed as RIP3-positive. Double staining of TUNEL and RIP3 demonstrated that TUNEL-positive cells exhibiting shrunken or fragmented nuclei, as generally observed in apoptotic cells, rarely expressed RIP3.

**Conclusions:**

The present study first demonstrated that the expression of RIP3 is dramatically upregulated in various neural cells in the injured spinal cord and peaked at 3 days after injury. Additionally, most of the PI-labeled cells expressed RIP3 in response to neural tissue damage after SCI. The present study suggested that the upregulation of the RIP3 expression may play a role as a novel molecular mechanism in secondary neural tissue damage following SCI. However, further study is needed to clarify the specific molecular mechanism underlying the relationship between the RIP3 expression and cell death in the injured spinal cord.

## Background

Necrosis was originally thought to be a purely passive and uncontrolled type of cell death. Necrosis, which is marked by cell swelling and membrane rupture, leads to inflammation via the release of intracellular signals [1–3]. In contrast, apoptosis, which is characterized by the activation of caspases and DNA fragmentation, was once considered the sole form of programmed cell death. Chromatin condensation, nuclear shrinkage and fragmentation and membrane blebbing are the result of the proteolytic activity of caspases and define the morphological characteristics of apoptosis [3–5].

“Necroptosis” is a newly identified type of programmed necrosis. Necroptosis is another form of programmed cell death that is regulated by the caspase-independent pathway and exhibits the morphological features of necrosis [6, 7]. Recent studies have shown that the receptor-interacting protein (RIP) family is specifically involved in regulating necroptosis [3]. RIP3, a member of the RIP family, is known to be a key mediator of necroptosis [8, 9]. RIP3 acts a nucleocytoplasmic shuttling protein and can locate not only in the cytoplasm but nucleus [10]. Various stimuli to cells can induce the formation of necrotic complexes that contain RIP1, TRADD, FADD and caspase-8. The interaction of RIP3 with RIP1 in the necrotic complex is an important step required for the execution of necroptosis [3, 11]. Previous studies have demonstrated that the RIP3 expression correlates with the induction of necroptosis in various types of cells [12–18]. RIP3 alone can trigger necroptosis, even in the absence of RIP1 [12, 14, 19]. Therefore, RIP3 is indispensable for necroptosis, whereas RIP1 may participate only in certain stimulus-induced types of cell necroptosis [9].

Furthermore, recent studies have shown an increased expression of RIP3 in lesions and the induction of necroptosis in various disease models [18, 20], such as that involving liver injury [14], ileitis [21], skin inflammation [22], and kidney ischemia–reperfusion injury [23]. The expression of RIP3 is also increased in retinal neurons in response to acute ischemic insults [24], and the upregulation of RIP3 contributes to the induction of necroptotic cell death in hippocampal neurons following cerebral ischemia [17].

Currently, it is considered that secondary damage after spinal cord injury (SCI) is caused by apoptosis, and most previous research related to cell death in the injured spinal cord has focused on apoptosis, not necroptosis.

In the present study, we investigated alterations in the RIP3 protein expression and the involvement of necroptosis in SCI using a spinal cord hemisection model in mice.

## Methods

### Animals

All experimental procedures were approved by the Institutional Animal Care and Use Committee of Tohoku University. All efforts were made to minimize the number of animals used and to decrease their suffering. Adult female C57BL/6J mice (8–10 weeks old; Charles River, Japan Inc., Yokohama, Japan) were used in this study. The mice were housed three or four per cage and kept at a temperature of 24 °C with free access to food and water before and after surgery.

### Spinal cord hemisection model

The mice were anesthetized with 2 % sevoflurane. A 15-mm midline skin incision was made, and the laminae of T9-11 were exposed. Laminectomy was performed at the thoracic vertebra at T10, exposing the dorsal surface of the spinal cord without disrupting the dura mater. With a sharp scalpel, the spinal cord was hemi-transected on the left side only [25–28]. In mice with a compromised bladder function (a rare complication), the bladder was manual expressed twice a day until spontaneous voiding was noted. The sham-operated animals underwent the same surgical procedures, although hemisection was not applied to the spinal cord. During the surgery, the rectal temperature was monitored and maintained at 37.0 ± 0.5 °C with a heating pad.

### Tissue preparation

At different time points (4 h, 24 h, 3, 7, and 21 days) after hemisection and immediately after the sham operation, the mice were overdosed via an intraperitoneal injection of 100 mg/kg sodium pentobarbital. The mice were then transcardially perfused with normal saline, followed by 4 % paraformaldehyde in 0.1 M PBS, pH 7.4. For immunohistochemical staining, the spinal cord segments containing the injured site were collected, postfixed in the same fixative overnight at 4 °C, cryoprotected in 30 % sucrose in PBS for 48 h at 4 °C and embedded in Optimal-Cutting-Temperature compound (Sakura Finetek, Japan). Serial 15-μm transverse cryostat sections obtained from around the injured site were mounted on slides. A total of 13 sequential sections were collected at 250-μm intervals that spanned 3000 μm in length along the spinal cord centered at the epicenter. The sections were used for both immunohistochemical and TUNEL staining, as described below.

### Immunohistochemical staining of RIP3

For the immunohistochemical staining of RIP3, the sections were washed in PBS for 15 min, after which they were washed with PBS containing 0.3 % Tween for 10 min and blocked with 3 % milk and 5 % FBS in 0.01 M PBS for 2 h. The sections were incubated with rabbit anti-RIP3 antibodies (1:100; Sigma-Aldrich) diluted in PBS overnight at 4 °C. After rinsing with PBS, the sections were incubated with secondary antibodies. The sections were coverslipped with Vectashield containing DAPI to label the nuclei (Vector Laboratories). In each experiment, the sections were stained at the same time.

### Counting and calculation of RIP3-positive cells

Following immunochemical staining of RIP3, as described above, each section was scanned using a fluorescence microscope (BX 51; Olympus, Tokyo, Japan). In order to quantify the RIP3 expression in the spinal cord, the total number of RIP3-positive cells in the injured and contralateral sides of the spinal cord was counted using the serial transverse sections at 250-μm intervals. The sections with the highest number of RIP3-positive cells on the injured side and the 250-μm rostral and caudal sections in each animal were selected. Then, the sum of the numbers in the three sections was compared between the injured side, the contralateral side and the sham group.

### Western blot analysis of RIP3

The mice were killed 3 days after spinal cord hemisection and the sham operation, and their spinal cords were removed. The spinal cord was homogenized in lysis buffer. The debris was removed via centrifugation, and the protein levels in the lysates were determined with the aid of a Bio-Rad protein assay (Bio-Rad Laboratories, USA). The protein (30 μg) in the lysates was resolved using SDS–polyacrylamide gel electrophoresis (PAGE) in 15 % gels and then electrophoretically transferred to a polyvinylidene difluoride membrane. The membranes were blocked for 1 h in TBST buffer (0.01 M Tris HCl, pH 7.5, 0.15 M NaCl and 0.05 % Tween 20) containing 3 % milk and incubated with rabbit anti-RIP3 antibodies (1:100; Santa Cruz Biotechnology, USA) diluted in TBST buffer overnight at 4 °C. The membranes were incubated with secondary antibodies linked to horseradish peroxidase (1:1000; Invitrogen). The immunoreactive bands were developed using the enhanced chemiluminescence reagent (Amersham Corp). The band density was quantified using a scanned densitometric analysis and the Image Lab software program version 4.1 (Bio-Rad Laboratories). The quantity of the band density was normalized according to the level of β-tubulin and compared between the injured and uninjured spinal cord samples.

### Double staining for RIP3 and various cell type markers

In order to examine the expression of RIP3 in a specific population of cells, the transverse sections obtained 3 days after hemisection were double-stained for RIP3 and various cell type markers: NeuN for neurons, GFAP for astrocytes and Olig2 for oligodendrocytes. The sections were incubated with a mixture of rabbit anti-RIP3 antibody (1:100; Sigma-Aldrich) and either goat anti-Olig2 (1:100; Santa Cruz Biotechnology), mouse anti-GFAP (1:50; Dako) or mouse anti-NeuN (1:100; Chemicon) antibodies diluted in PBS overnight at 4 °C. After rinsing with PBS, the sections were incubated with secondary antibodies, and then mounted with Vectashield containing DAPI to label the nuclei (Vector Laboratories). The specificity of the RIP3 antibody was evaluated by omission of the primary antibody in immunohistochemistry. Omitting the primary antibody (no primary immunoglobulin G control) in this protocol abrogated the staining, demonstrating the specificity of the immunoreactive staining [14, 24].

### RIP3 staining using propidium iodide-labeled spinal cord sections

In order to detect plasmalemma permeability, which is a hallmark of necrotic cell death, in the injured spinal cord at 3 days after hemisection, propidium iodide (PI) labeling was performed as previously described [29–31]. Briefly, PI (10 mg/mL; Sigma-Aldrich) was diluted in 0.1 mL of PBS and then intraperitoneally injected at a dose of 1 mg/kg body weight at 1 h before sacrifice. The mice were transcardially perfused, and the spinal cords were collected and sectioned as described above. For immunohistochemical detection of the RIP3 expression in the PI-labeled cells, the PI-labeled spinal cord transverse sections were stained for RIP3, as outlined above. Using a fluorescence microscope (BX 51; Olympus, Tokyo, Japan), the propidium iodide labeling was detected using emission and excitation wavelengths of 568 and 585 nm, respectively.

### Double staining of RIP3 and TUNEL

In order to identify DNA fragmentation in the cells expressing RIP3, double staining of RIP3 and terminal deoxynucleotidyl transferase-mediated dUTP nick end labeling (TUNEL) was performed using the transverse sections at 3 days after hemisection. Following immunohistochemical staining of RIP3, as described above, the TUNEL was performed using the *In Situ* Cell Death Detection Kit (Roche Diagnostics).

### Statistical analysis

Significant differences in the number of RIP3-positive cells and the band density of the Western blots were analyzed using the unpaired *t* test. In all analyses, a *p* value of <0.05 was considered to be statistically significant.

## Results

### Immunohistochemical staining for RIP3

**Fig. 1:**
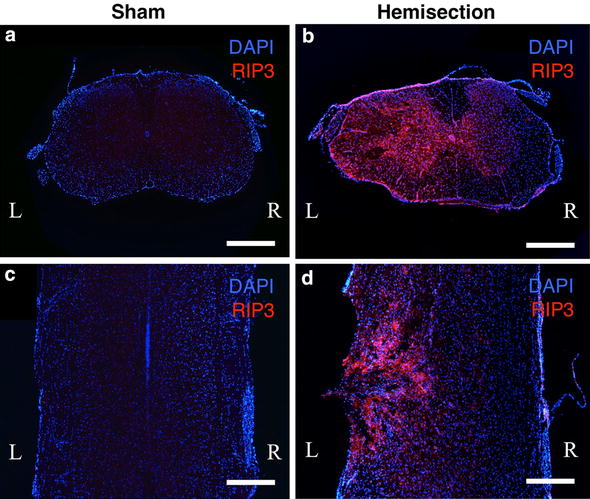
Immunohistochemical staining of RIP3 using spinal cord sections obtained 3 days after hemisection and the sham operation. The uninjured spinal cords in the sham operated mice (**a**, **c**) showed no obvious RIP3 expression. In contrast, a higher expression of RIP3 was observed on the injured side (*L*) than on the contralateral side (*R*) in the transverse (**b**) and coronal (**d**) sections after hemisection. *Scale bars* 500 μm

The number of cells expressing RIP3 was increased on the injured side at 3 days after spinal cord hemisection (Fig. 1). Cells expressing RIP3 were observed in both the gray and white matter of the injured side. However, all cells on the injured side did not express RIP3. On the contralateral side, the number of cells expressing RIP3 was obviously low, similar to that observed in the sham group.

### Time course of the RIP3 expression after hemisection

**Fig. 2:**
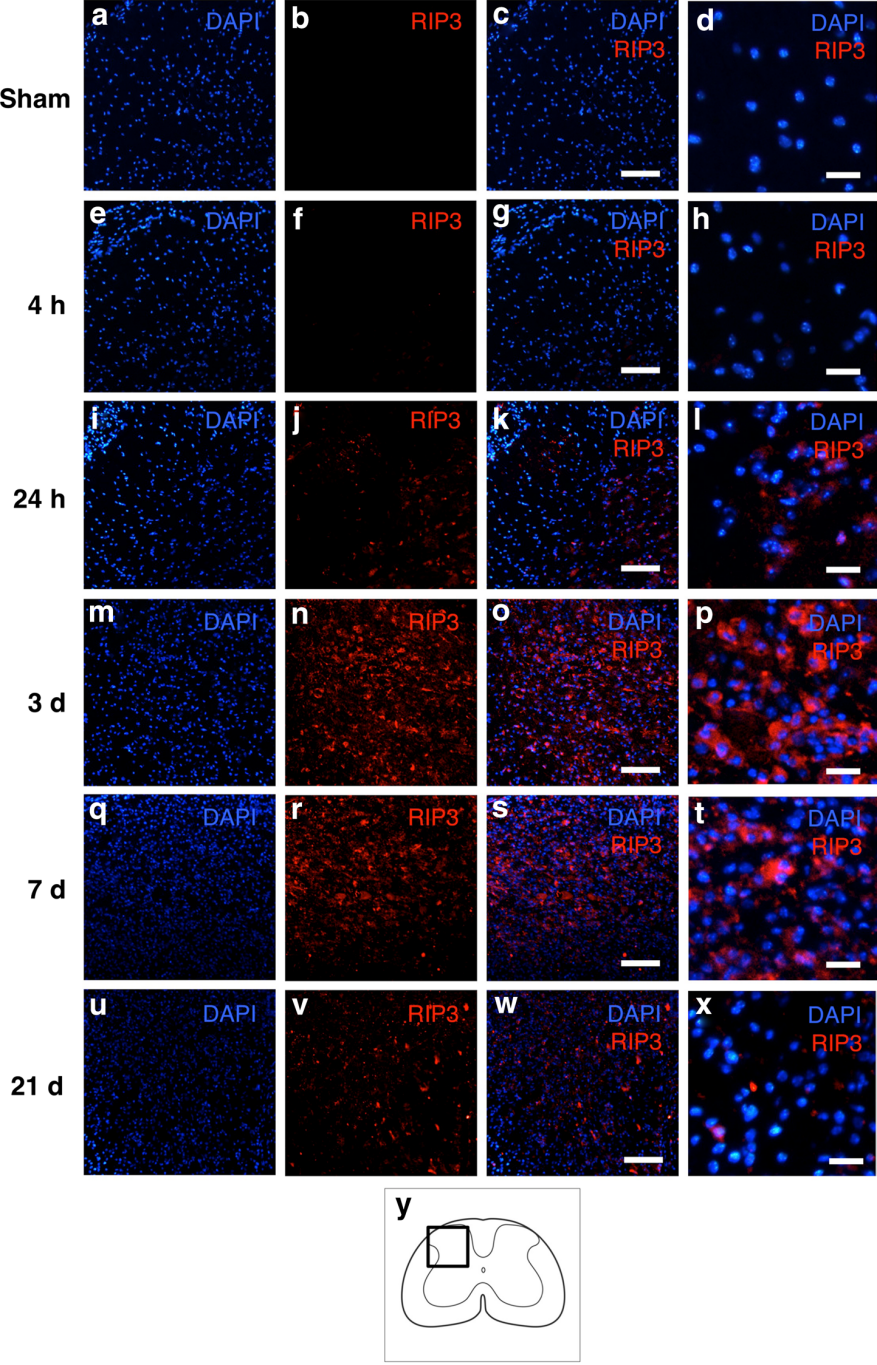
Immunohistochemical staining of RIP3 on the injured side in the transverse sections obtained at different time points. The expression of RIP3 was not observed at 4 h (**e**–**h**); however, it started to increase at 24 h (**i**–**l**), in comparison to that observed in the sham group (**a**–**d**). The population of RIP3-expressing cells was obviously larger at 3 and 7 days (**m**–**t**) than at the other time points. *Scale bars* 100 μm (**a**–**c**, **e**–**g**, **j**–**k**, **m**–**o**, **q**–**s**, **u**–**w**), 20 μm (**d**, **h**, **l**, **p**, **t**, **x**). The schematic drawing illustrates the location of the micrographs (**y**)

Representative pictures showed that the number of cells expressing RIP3 was increased on the injured side at each time point after hemisection compared to that observed in the sham group (Fig. 2). The population of cells expressing RIP3 on the injured side was relatively larger at 3 and 7 days compared to that noted at the other time points.

**Fig. 3:**
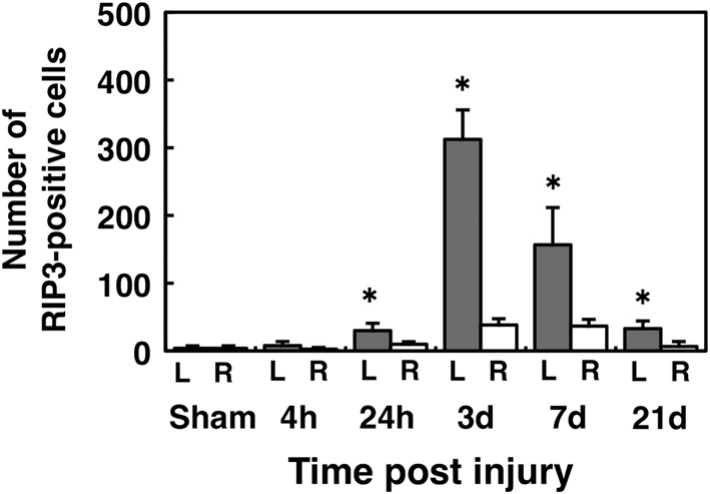
Number of RIP3-positive cells on the injured and contralateral sides at different time points. The number of RIP3-positive cells on the injured side (*L*) was significantly higher than that observed on the contralateral side (*R*) and in the sham group at 24 h and 3, 7 and 21 days. (*p < 0.05, n = 3 per each group). The values are presented as the mean ± SD

When counting the number of RIP3-positive cells (Fig. 3), those observed on the injured side were found to be significantly higher than those seen on the contralateral side and in the sham control group. A significant increase in the number of RIP3-positive cells was first noted at 24 h and lasted for at least 21 days (*P* < 0.05). The maximum number of RIP3-positive cells on the injured side was observed at 3 days after hemisection.

### Western blot analysis of RIP3

**Fig. 4:**
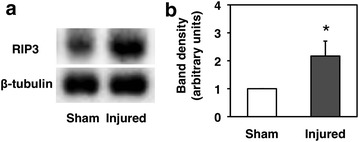
RIP3 protein expression at 3 days after hemisection. **a** Western blotting showed that the RIP3 protein expression in the injured spinal cord was obviously increased compared to that observed in the uninjured spinal cord after the sham operation. **b** A quantitative analysis of the Western blots showed that the level of RIP3 proteins in the injured spinal cord was significantly higher than that observed in the sham control samples (*p < 0.05, n = 5 per each group). The quantity of the band density was normalized according to the level of β-tubulin. The values are presented as the mean ± SD

In the Western blot analysis, the level of RIP3 proteins was significantly higher in the injured spinal cord than in the uninjured spinal cord (Fig. 4a). In the analysis of the band density, the RIP3 expression was 2.2 ± 0.5-fold higher in the injured spinal cord (Fig. 4b, *P* = 0.005).

### Double staining of RIP3 and various cell type markers

**Fig. 5:**
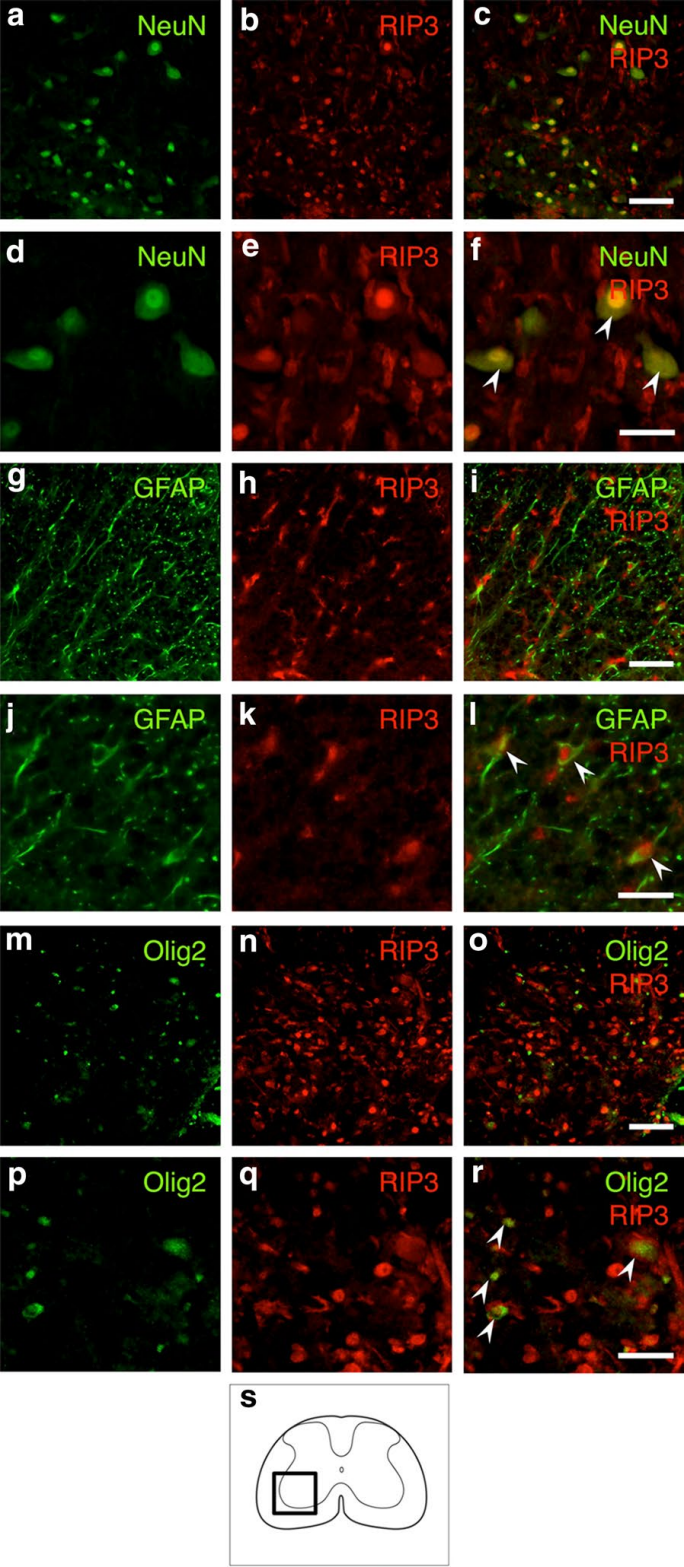
Double staining for RIP3 and various cell type markers on the injured side in the transverse sections after hemisection. The RIP3 expression was observed in the NeuN-, GFAP- and Olig2-labeled cells (*arrowheads*), demonstrating that the RIP3 expression was increased in neurons, astrocytes and oligodendrocytes, respectively. *Scale bars*: 100 μm (**a**–**c**, **g**–**i**, **m**–**o**), 50 μm (**d**–**f**, **j**–**l**, **p**–**r**). The schematic drawing illustrates the location of the micrographs (**s**)

In order to examine the RIP3 expression in specific populations of cells, the transverse sections obtained at 3 days after hemisection were double-stained for RIP3 and various neural cell type markers. On the double staining, the expression of RIP3 was observed in the NeuN-, GFAP- and Olig2-labeled cells (Fig. 5). The double staining demonstrated the RIP3 expression in neurons, astrocytes and oligodendrocytes. However, not all of these cells expressed RIP3.

### RIP3 expression in the PI-labeled cells

**Fig. 6:**
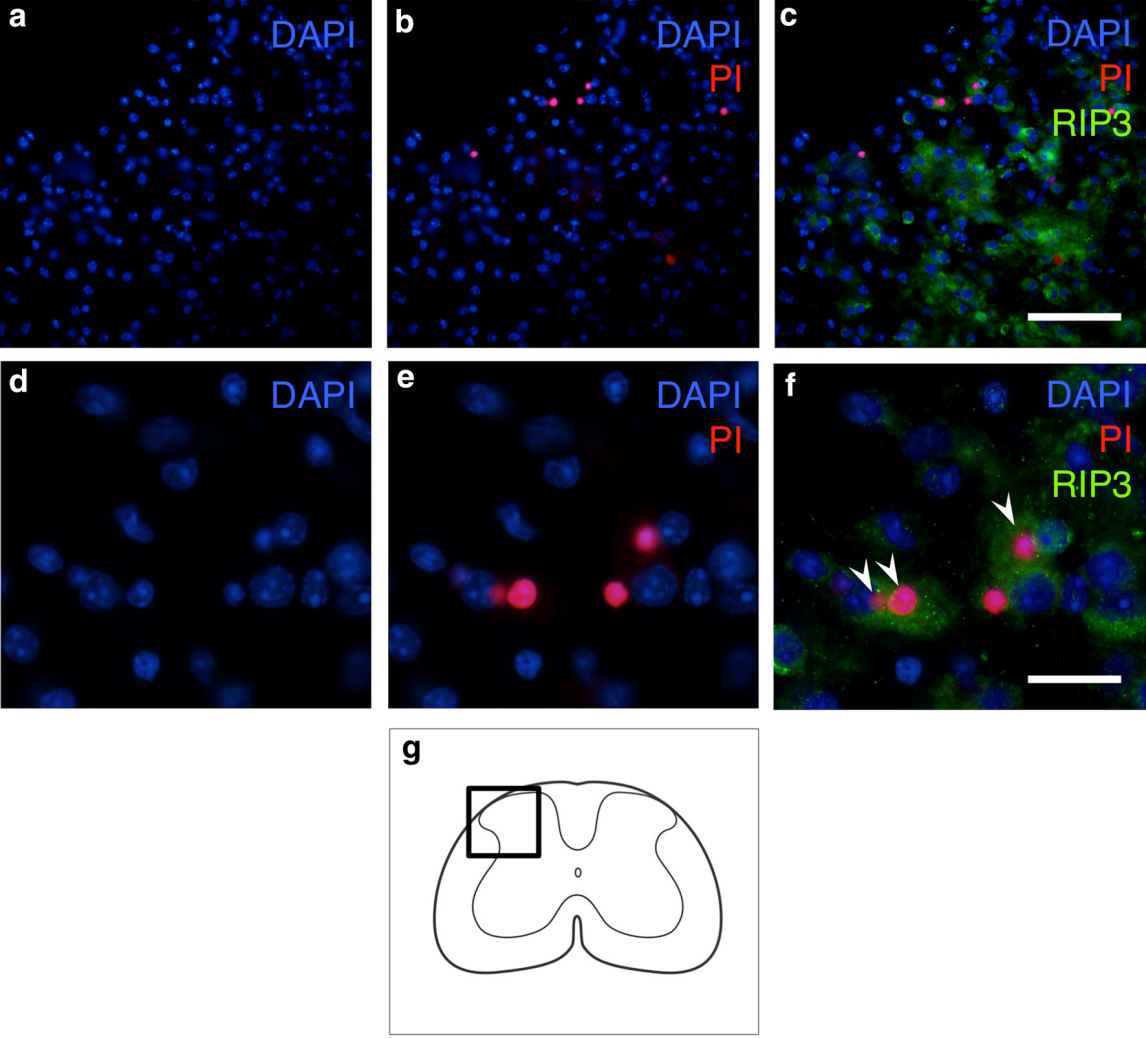
Immunostaining for RIP3 using propidium iodide (PI)-labeled sections obtained 3 days after hemisection. The representative pictures show that the PI-labeled cells were frequently observed to be RIP3-positive (**a**–**c**). At higher magnification (**d**–**f**), most of the nuclei in the PI-labeled cells expressing RIP3 (*arrowheads* in **f**) were round, which is normally observed in cells undergoing necrotic cell death, not fragmented or shrunken, as observed in apoptotic cells. *Scale bars* 100 μm (**a**–**c**), 20 μm (**d**–**f**). The schematic drawing illustrates the location of the micrographs (**g**)

Plasmalemma permeability, a hallmark of necrotic cell death, including necroptosis, was detected using PI-labeling in vivo. In RIP3 immunostaining using PI-labeled spinal cord sections. The number of both PI-labeled cells and RIP3-expressing cells increased on the injured side, and the PI-labeled cells were frequently observed to be RIP3-positive (Fig. 6). At a higher magnification, most of the nuclei in the PI-labeled cells expressing RIP3 were round, as generally observed in necrotic cell death.

### Double staining of RIP3 and TUNEL

**Fig. 7:**
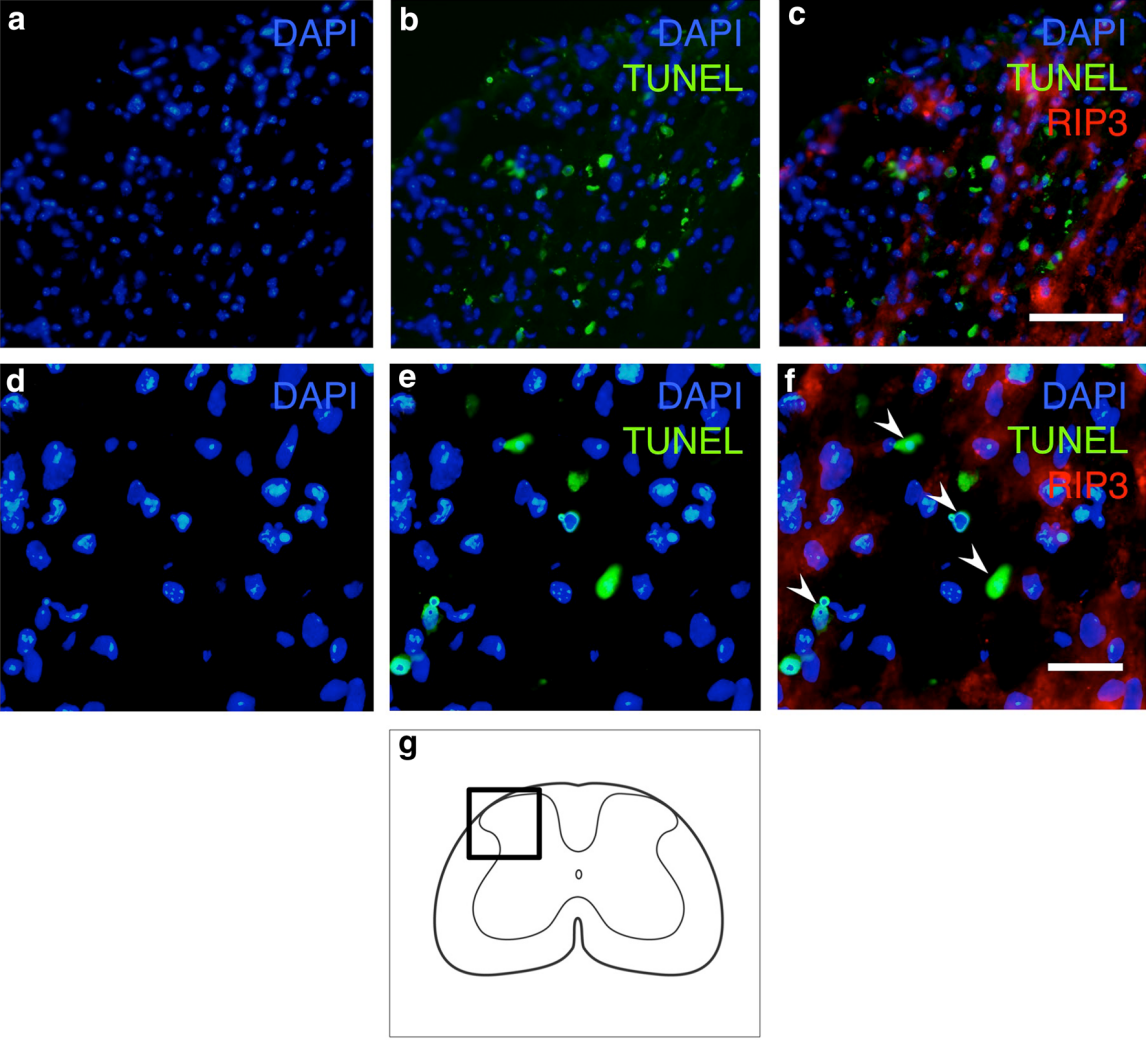
Double staining for RIP3 and TUNEL on the injured side in transverse sections obtained 3 days after hemisection. The numbers of RIP3-expressing and TUNEL-positive cells were obviously increased in the injured spinal cord (**a**–**c**). At higher magnification (**d**–**f**), the TUNEL-positive cells exhibiting shrunken or fragmented nuclei (*arrowheads*), a hallmark of apoptotic cell death, rarely expressed RIP3. *Scale bars* 100 μm (**a**–**c**), 20 μm (**d**–**f**). The schematic drawing illustrates the location of the micrographs (**g**)

In order to detect DNA fragmentation in the cells expressing RIP3, we performed double staining of RIP3 and TUNEL. On the injured side, the numbers of TUNEL-positive and RIP3-positive cells were observed to have obviously increased (Fig. 7a–c). However, the RIP3 expression was rarely observed in the TUNEL-positive cells. Under higher magnification, most of the TUNEL-positive cells exhibiting shrunken or fragmented nuclei, as is typical of apoptotic cells, did not express RIP3 (Fig. 7d–f).

## Discussions

In the present study, the level of RIP3 proteins significantly increased on the injured site after spinal cord hemisection. An increase in the number of RIP3-positive cells at the injured site was observed starting at 24 h, peaked at 3 days and lasted for at least 21 days after injury. The RIP3 expression was observed in neurons, astrocytes and oligodendrocytes. Most of the PI-labeled cells expressed RIP3 in response to neural tissue damage after SCI. These results suggest that the expression of RIP3 was dramatically upregulated in various neural cells and may be involved in the pathological mechanism at the lesion site following SCI.

Traditionally, secondary damage of neural tissue following SCI has been considered to be caused by apoptosis, not necroptosis. Most previous research related to cell death in the injured spinal cord has focused on apoptosis. In the present study, both immunohistochemistry and Western blot analysis demonstrated that the RIP3 expression was significantly upregulated at the lesion site after SCI. Importantly, the upregulation of the RIP3 protein was observed starting at 24 h, after which it peaked at 3 days and lasted for 21 days after injury. The time course of the RIP3 expression is quite similar to that of secondary damage following SCI [32–34]. Additionally, we have previously confirmed that the number of TUNEL-positive cells peaked at 3 days in the thoracic spinal cord hemisection model in mice (data not shown). The TUNEL-positivity potentially indicates apoptotic cell death as well as necrotic cell death if the morphological characteristics of the cells are ignored [35]. Whalen et al. [31] suggested that different phenotypes of cell death can occur at the lesion site after the CNS injury. These findings suggest that the increased expression of RIP3 in the injured spinal cord may contribute to secondary neural tissue damage and is possibly associated with not only apoptosis, but also necrotic cell death after SCI.

Recent studies have demonstrated that necroptosis plays a role in various pathological conditions in the CNS. Necroptosis can contribute to neural tissue damage in the model of adult brain ischemia [17, 36, 37], neonatal hypoxia–ischemia [38], traumatic brain injury [29] and neurodegenerative diseases [39, 40]. Retinal ischemia [24, 41] and photoreceptor loss-associated retinal disorders [42] also involve neuronal necroptosis. Previous studies have shown that apoptosis is associated with peculiar morphological traits, including chromatin condensation and nuclear shrinkage and fragmentation, as well as blebbing of the intact plasma membrane and shedding of vacuoles containing cytoplasmic portions known as apoptotic bodies [1, 4, 5]. On the other hand, necroptosis involves a similar morphology to that of necrosis, such as minor ultrastructural modifications of the nucleus (dilatation of the nuclear membrane), osmotic swelling of organelles, an increased cell volume and breakdown of the plasma membrane [3, 11]. In the present study, most of the PI-positive cells expressed RIP3 in the injured spinal cord and contained round nuclei, which is generally observed in cells undergoing necrotic cell death. Additionally, the TUNEL-positive cells exhibiting shrunken or fragmented nuclei rarely expressed RIP3 at the lesion site. Based on the increased expression of RIP3 and the morphology of the nuclei in these cells, necroptosis may be one of the several types of cell deaths occurring in the injured spinal cord.

However, it has been reported that the PI- or TUNEL-positivity cannot perfectly differentiate between necrotic and apoptotic cells. A previous study suggested that PI-positive cells can occasionally be TUNEL-positive after CNS injury [31]. The PI-positivity is a hallmark of necrosis, but cannot be used by itself to identify necrotic cells because PI can enter cells with activated pannexin channels [43]. The TUNEL-positivity potentially indicates apoptotic cell death as well as necrotic cell death [35]. Additionally, the PI- or TUNEL-positivity did not perfectly reflect the RIP3 expression in the lesion site after hemisection in this study. Thus, from the data presented in this study, no firm conclusion about cell death in the RIP3-positive cells in the injured spinal cord can be made. The precise molecular mechanism underlying the relationship between RIP3 expression and PI- and/or TUNEL-positivity in neural tissue damage requires further clarification.

Previous studies have suggested that necroptosis is induced in various neural cells. The RIP3 expression in retinal neurons is upregulated in response to acute ischemic insults [24], and cerebral ischemia induces necroptotic cell death in hippocampal neurons [17]. In addition, necroptosis drives motor neuron death in amyotrophic lateral sclerosis ALS [40], Furthermore, hemin induces necroptotic cell death in cortical astrocyte cultures [44]. Necroptosis is induced in cultured rat astrocytes by the administration of staurosporine [45] Necroptosis contributes to arachidonic acid-induced oxidative cell death in primary oligodendrocyte precursor cultures [46]. According to our results, the expression of RIP3 is observed in neurons, astrocytes and oligodendrocytes in the injured spinal cord. These findings suggested that necroptosis may occur in various neural cells and may contribute to multiple pathological mechanisms after SCI. However, the molecular mechanisms defining the features of necroptosis are not fully understood, and the precise pathological mechanisms induced by necroptosis after SCI remain unclear. Further studies to clarify the molecular and biological mechanisms of necroptosis will hopefully lead to the development of novel therapeutic strategies for the treatment of SCI.

## Conclusion

In the present study, the expression of RIP3 is dramatically increased at the lesion site after SCI. The increased RIP3 expression was observed in neurons, astrocytes and oligodendrocytes. Most of the PI-labeled cells expressed RIP3 in response to neural tissue damage. This study is the first to provide evidence supporting the increased expression of RIP3 involved in neural tissue damage after SCI.
